# Misplaced left internal jugular venous catheter with an exceptional location

**DOI:** 10.11604/pamj.2016.24.305.10424

**Published:** 2016-08-10

**Authors:** Hassen Ben Ghezala, Najla Feriani

**Affiliations:** 1 Service Universitaire des Urgences et de Réanimation Médicale, Hôpital Régional de Zaghouan, Faculté de Médecine de Tunis, Tunisie; 2 Service Universitaire de Chirurgie Générale, Hôpital Régional de Zaghouan, Faculté de Médecine de Tunis, Tunisie

**Keywords:** Internal jugular vein, central vein catheter, intensive care

## Image in medicine

Large numbers of central venous catheters (CVCs) are placed each year in the intensive care units and misplacement occurs frequently. Many critically ill patients require central venous catheterization for multiple and varied reasons. Internal jugular vein (IJV) catheter is one of the most frequent central venous catheters in intensive care units not only in Africa but all over the word. The right position of the catheter should be always verified by chest X ray after each catheterization even when it is ultrasound-guided. Acquired abnormalities such as stenosis or thrombosis of the central veins can be problematic. Catheters can also be misplaced outside veins in a patient with otherwise normal anatomy with potentially disastrous consequences. We report in this work an exceptional image of chest X ray showing an extremely rare misplacement of an internal jugular vein central catheter. This occurred in a 73-years-old man admitted to our intensive care unit for an acute respiratory failure due to right pulmonary complex contusions due to a traffic accident which required intubation and mechanical ventilation. The chest X ray presented in this work shows an axillary location of the central catheter. It was documented that the catheter was inserted into the left axillary vein. Interestingly, there was no problem for infusion of fluids via this catheter. However, we removed the catheter without further complications and IJV cannulation was performed successfully via left. With regard to misplaced CVCs, in the short term, a useful aide memoir is: “if in doubt, don't take it out”.

**Figure 1:**
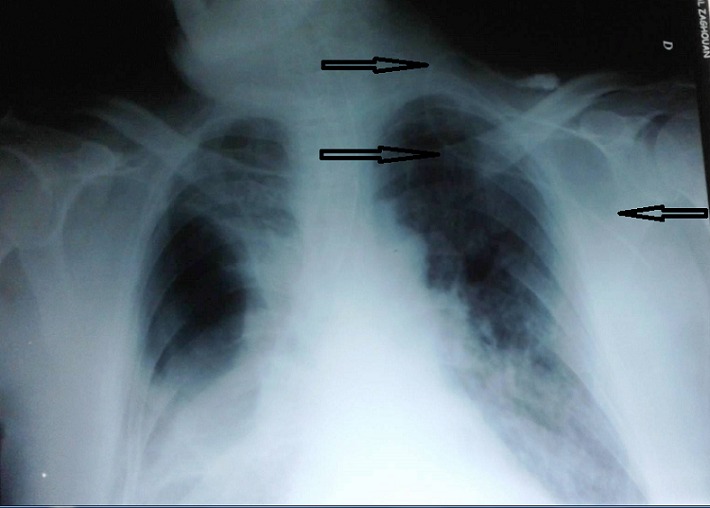
Chest X ray showing an aberrant position of a left internal jugular vein catheter in the left axillary vein

